# Promoting Effect of Cerium Oxide on the Catalytic Performance of Yttrium Oxide for Oxidative Coupling of Methane

**DOI:** 10.3389/fchem.2018.00581

**Published:** 2018-11-22

**Authors:** Masaaki Haneda, Yuya Katsuragawa, Yuichiro Nakamura, Atsuya Towata

**Affiliations:** ^1^Advanced Ceramics Research Center, Nagoya Institute of Technology, Tajimi, Japan; ^2^Frontier Research Institute for Materials Science, Nagoya Institute of Technology, Nagoya, Japan; ^3^Magnetic Powder Metallurgy Research Center, National Institute of Advanced Industrial Science and Technology, Nagoya, Japan

**Keywords:** oxidative coupling of methane, cerium oxide, yttrium oxide, ^16^O/^18^O isotopic exchange, H_2_-TPR

## Abstract

The promoting effect of CeO_2_ on the catalytic performance of Y_2_O_3_, which is moderately active catalyst, for the oxidative coupling of methane (OCM) reaction was investigated. The addition of CeO_2_ into Y_2_O_3_ by coprecipitation method caused a significant increase in not only CH_4_ conversion but also C_2_ (C_2_H_6_/C_2_H_4_) selectivity in the OCM reaction. C_2_ yield at 750 °C was increased from 5.6% on Y_2_O_3_ to 10.2% on 3 mol% CeO_2_/Y_2_O_3_. Further increase in the CeO_2_ loading caused an increase in non-selective oxidation of CH_4_ to CO_2_. A good correlation between the catalytic activity for the OCM reaction and the amount of H_2_ consumption for the reduction of surface/subsurface oxygen species in the H_2_-TPR profile was observed, suggesting the possibility that highly dispersed CeO_2_ particles act as catalytically active sites in the OCM reaction. The ^16^O/^18^O isotopic exchange reaction suggested that the beneficial role of CeO_2_ in the OCM reaction is to promote the formation of active oxygen species *via* the simple hetero-exchange mechanism, resulting in the promotion of CH_4_ activation.

## Introduction

Methane, which is the main constituent of natural gas, is an abundant hydrocarbon resource for energy and chemicals. Since methane is the most stable hydrocarbon, the selective oxidation to the chemicals seems to be difficult compared with other hydrocarbons. Therefore, in many cases, methane is first partially oxidized to synthesis gas (CO + H_2_), and then converted to the chemicals such as methanol, acetic acid, light hydrocarbons and so on. This is well-known process as C1 chemistry.

On the other hand, as for the direct conversion of methane to the chemicals, oxidative coupling of methane (OCM) is regarded as energy efficient and simple chemical process for the production of higher hydrocarbons, especially, ethane and ethylene (Lunsford, 1995). The development of highly active OCM catalyst has been continuously performed over the past few decades (Hutchings et al., 1989; Arndt et al., 2011; Takanabe, 2012; Farrell et al., 2016) since the pioneering works of Keller and Bhasin (1982). The OCM reaction proceeds *via* the formation of methyl radicals formed by the reaction of methane with active oxygen species and subsequent coupling the methyl radicals (Dubois and Cameron, 1990). Therefore, designing the catalyst surface to create the active oxygen species such as O^−^, O${}_{2}^{2-}$, and O^2−^ for the OCM reaction must be the important strategy to develop highly active catalysts (Kumar et al., 2016).

Cerium oxide (CeO_2_) is well-known material showing reversible oxygen release/absorb properties, so-called OSC (oxygen storage capacity) (Trovarelli, 1996), leading us to the expectation that CeO_2_ can promote the activation of methane and then improve the catalytic performance for the OCM reaction. The promoting effect of CeO_2_ on the OCM reaction has already been reported by many researchers. For example, Tang et al. (2011) reported that the addition of CeO_2_ into Li/MgO caused an increase in the CH_4_ conversion at lower reaction temperature with little change in C_2_ selectivity. They explained the promoting effect of CeO_2_ by oxygen activation caused *via* the electron transfer between Ce^4+^/Ce^3+^ and Li/MgO. A similar promoting effect of CeO_2_ was reported for Na/CaO (Pacheco Filho et al., 2000) and Li/MgO (Elkins et al., 2016). In case of alkali-doped catalysts, Li^+^O^−^ and Na^+^O^−^ sites are proposed to participate in oxygen activation (Ito et al., 1985; Lin et al., 1986). Therefore, the role of CeO_2_ is suspected to improve the catalytic ability of Li^+^O^−^ and Na^+^O^−^ sites through the electronic interaction. On the other hand, Rane et al. (2006) investigated the additive effect of rare earth elements (La, Sm, Ce, Nd, and Yb) on the OCM activity of CaO, and found that CeO_2_ is not good additive for the formation of C_2_ products. This is probably due to the high ability of CeO_2_ for the complete oxidation of CH_4_ to CO_2_ (Ferreira et al., 2012; Xu et al., 2018).

Recently, we have investigated that similarity of catalytically active sites for oxidative coupling of methane (OCM) and NO decomposition, and found that the catalysts showing the activity for the latter reaction are also active for the former reaction (Haneda et al., 2018). We also reported that the addition of small amount of CeO_2_ into Ba/Y_2_O_3_ causes a significant increase in the NO decomposition activity (Doi et al., 2015), suggesting the possibility that CeO_2_ behaves as effective promoter for the OCM reaction. In this study, we prepared CeO_2_-promoted Y_2_O_3_ as the OCM catalyst by various methods, and found that CeO_2_/Y_2_O_3_ prepared by coprecipitation method showed the highest OCM activity. The active state and the role of CeO_2_ in the OCM reaction are discussed on the basis of its catalyst characterizations.

## Experimental

### Catalyst preparation

A plural CeO_2_-promoted Y_2_O_3_ catalyst was prepared by coprecipitation, homogeneous precipitation, impregnation and hydrothermal methods. The loading of CeO_2_ was fixed at 3 mol%, except for the case of coprecipitation method. The details of CeO_2_/Y_2_O_3_ catalysts as well as Y_2_O_3_ itself prepared in the present study are summarized in Table 1.

**Table 1:** Physico-chemical properties of CeO_2_/Y_2_O_3_.

	**Preparation method**	**Crystallite size^a^ (nm)**	**BET surface area (m^2^g^−1^)**
Y_2_O_3_	Precipitation using NH_3_	40.0	23.9
CeO_2_/Y_2_O_3_(CP)	Coprecipitation using NH_3_	25.1	26.3
CeO_2_/Y_2_O_3_(HP)	Homogeneous precipitation using urea	25.3	27.5
CeO_2_/Y_2_O_3_(I)	Impregnation	33.3	23.0
CeO_2_/Y_2_O_3_(HT)	Hydrothermal at 200 °C	9.7	13.0

a *The crystallite size of Y_2_O_3_ was calculated from the XRD peak, given in Figure 1, from the (222) plane using Scherrer's equation*.

#### Yttrium oxide (Y_2_O_3_)

Y_2_O_3_ was prepared by precipitation method using yttrium(III) nitrate and ammonia aqueous solution, followed by drying and calcination at 800°C for 5 h in air.

#### Coprecipitation method [CeO_2_/Y_2_O_3_(CP)]

An aqueous solution of ammonia (10% NH_4_OH, FUJIFILM Wako Pure Chemical Corporation) as a precipitation agent was added to an aqueous solution of yttrium(III) nitrate [Y(NO_3_)_3_·6H_2_O, FUJIFILM Wako Pure Chemical Corporation], and ammonium cerium(IV) nitrate [(NH_4_)_2_Ce(NO_3_)_6_, FUJIFILM Wako Pure Chemical Corporation] at room temperature. The precipitate thus obtained was washed with distilled water, followed by drying and calcination at 800°C for 5 h in air. The loading of CeO_2_ was changed from 1 to 10 mol%. The samples are abbreviated as CeO_2_(*x*)/Y_2_O_3_(CP), where *x* is the loading of CeO_2_.

#### Homogeneous precipitation method [CeO_2_/Y_2_O_3_(HP)]

The precipitation of yttrium and cerium hydroxides was obtained by adding urea [CO(NH_2_)_2_, FUJIFILM Wako Pure Chemical Corporation] to an aqueous solution of yttrium(III) nitrate and cerium(III) nitrate [Ce(NO_3_)_3_·6H_2_O, FUJIFILM Wako Pure Chemical Corporation] and stirring at 90°C for 24 h, and then washed with distilled water, followed by drying and calcination at 800°C for 5 h in air.

#### Impregnation method [CeO_2_/Y_2_O_3_(I)]

The deposition of Ce ions onto Y_2_O_3_ was carried out by impregnating Y_2_O_3_ powder, which was prepared by precipitation method as mentioned above, with an aqueous solution of cerium(III) nitrate, followed by drying and calcination at 800°C for 5 h in air.

#### Hydrothermal method [CeO_2_/Y_2_O_3_(HT)]

The oleate solution prepared by dissolving potassium oleate (C_17_H_33_COOK, 19% solution, FUJIFILM Wako Pure Chemical Corporation) with distilled water was added to an aqueous solution of yttrium(III) nitrate and ammonium cerium(IV) nitrate at room temperature under vigorously-stirred condition, followed by addition of ammonia aqueous solution. The mixture solution thus obtained was transferred to a Teflon vessel and then treated at 200°C for 6 h in an autoclave. The product thus obtained was washed with distilled water, followed by drying and calcination at 800°C for 5 h in air.

### Catalyst characterizations

X-ray diffraction (XRD) patterns were recorded using a Rigaku MiniFlex diffractometer with Cu Kα radiation at 30 kV and 15 mA. The scanning was done from 2θ = 15–65° at a speed of 1 deg min^−1^. The BET surface area of the catalysts was determined by N_2_ physisorption at liquid nitrogen temperature using a BELSORP mini-II, after evacuating the samples at 300°C for 1 h. Raman spectra were measured with a Micro-RAM300/NK (Lambda Vision) equipped with a TE-cooled charge coupled device (CCD) detector and a green laser (λ = 532 nm) under the ambient atmosphere. Direct observation of CeO_2_/Y_2_O_3_(CP) samples by TEM was performed with a JEM-2100 (JEOL) operating at an acceleration voltage of 200 kV. H_2_-TPR measurement was conducted to estimate the reducibility of the catalysts (Haneda et al., 2015). The TPR profiles were obtained from room temperature to 800°C in a 30 cm^3^min^−1^ flow of 5% H_2_/Ar at a heating rate of 10 °C min^−1^. The consumption of H_2_ was monitored using a thermal conductivity detector (TCD).

The ^16^O/^18^O isotopic exchange reaction was carried out in a flow reactor system. An adequate amount of the sample (30 mg) was installed in a quartz reactor, and then pretreated in a flow of 5% O_2_/He at 600°C for 1 h. After cooling to 100°C in flowing 5% O_2_/He, the sample was purged with He at 100°C for 30 min. The reaction gas composed of 1% ^18^O_2_/He was fed to the catalyst at a rate of 20 cm^3^ min^−1^, and then the temperature was increased up to 600 °C at a heating rate of 10°C min^−1^. The masses of 32 (^16^O_2_), 34 (^18^O^16^O), and 36 (^18^O_2_) were continuously monitored by a quadrupole mass spectrometer (PFEFFER OminiStar).

### Activity measurement

The catalytic activity for oxidative coupling of methane was evaluated using a fixed-bed continuous flow reactor. The reaction gas composed of 44.4% CH_4_ and 11.1% O_2_ diluted with He as the balance gas was fed to a 0.1 g catalyst that had been pretreated *in situ* in a flow of He at 650°C for 2 h at a rate of 45 cm^3^ min^−1^. The effect of variation of CH_4_/O_2_ ratio was also investigated, where O_2_ concentration was varied over the range of 5.5–22.2% and CH_4_ concentration was fixed at 44.4%. The reaction temperature was increased from 650 to 800°C in steps of 50°C, and the steady-state catalytic activity was measured at each temperature. The effluent gas was analyzed with the use of on-line gas chromatograph equipped with TCD and FID as a detector (Shimadzu GC-2014ATTF) using a Molecular Sieve 13X column (for analysis of O_2_, CH_4_, and CO), a Porapak QS column (for analysis of CO_2_), and a DC-200 (20%)/Shimalite column (for analysis of C_2_H_4_ and C_2_H_6_).

## Results and discussion

### Activity of CeO_2_/Y_2_O_3_ prepared by different method

Figure 1A shows the XRD patterns of 3 mol% CeO_2_/Y_2_O_3_ prepared by different method. Distinct XRD peaks indexed to the cubic phase of Y_2_O_3_ were observed for all the samples. No peaks due to CeO_2_ were detected because of the low loading (3 mol%), suggesting the presence of CeO_2_ nanoparticles. It is of interest that a shift of XRD peaks due to Y_2_O_3_ toward lower angle was observed for CeO_2_/Y_2_O_3_(HP) (Figure 1B–b), suggesting the formation of CeO_2_-Y_2_O_3_ solid solution. This can be explained by the fact that the radius of Ce^3+^ ion (0.114 nm)/Ce^4+^ ion (0.097 nm) is larger than that of Y^3+^ ion (0.09 nm) (Shannon, 1976). Homogeneous precipitation including Ce^3+^ and Y^3+^ ions may be produced under the conditions, where the pH of solution is gradually increased *via* the hydrolysis of urea.

**Figure 1:**
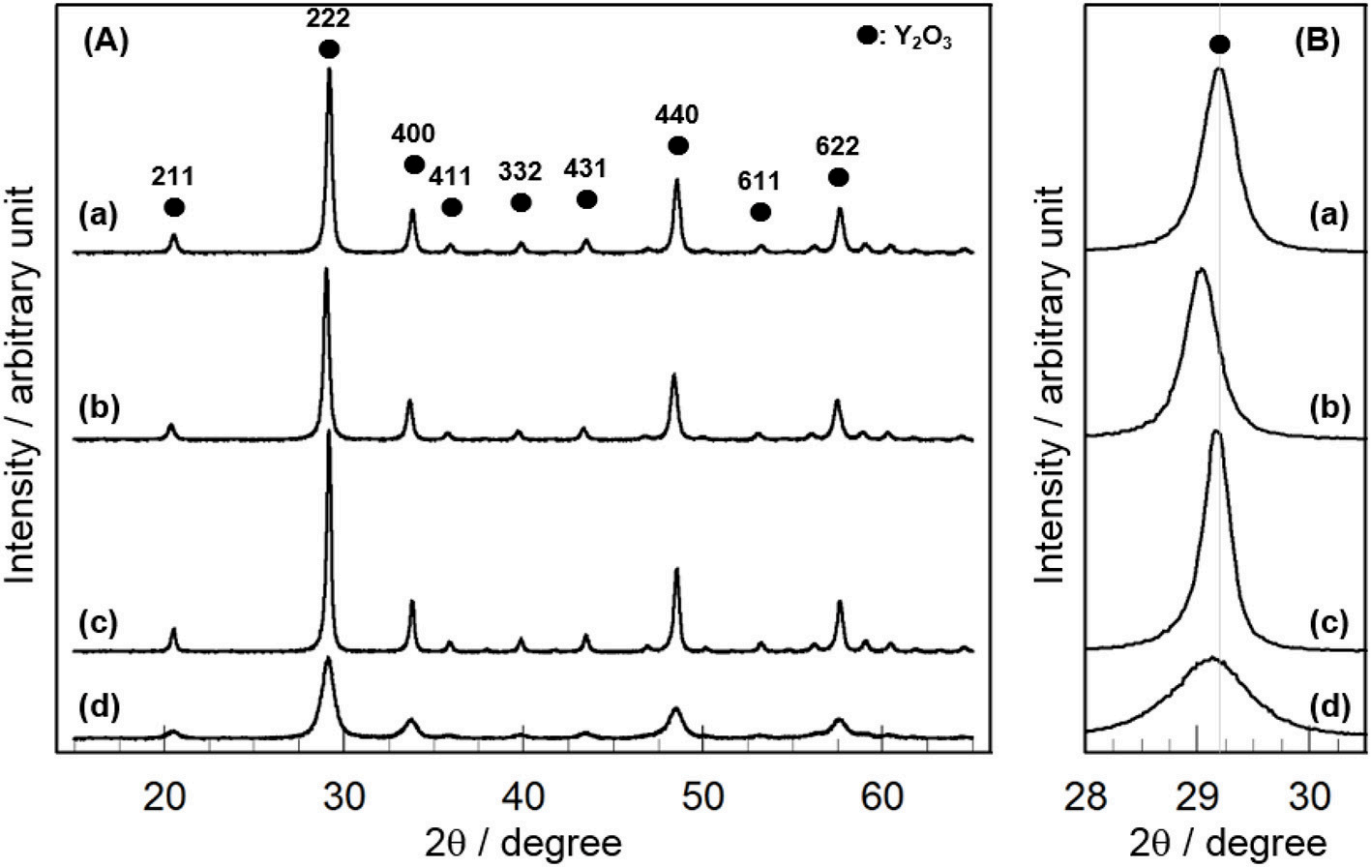
XRD patterns of (a) CeO_2_/Y_2_O_3_(CP), (b) CeO_2_/Y_2_O_3_(HP), (c) CeO_2_/Y_2_O_3_(I), (d) CeO_2_/Y_2_O_3_(HT) given in the wide range of 2θ = 16 − 64° **(A)** and in the expanded range of 2θ = 28.0 − 30.5° **(B)**.

Table 1 summarizes the crystallite size of Y_2_O_3_, which was calculated from the XRD peak due to the (222) plane (2θ = 29.1°) using Scherrer's equation without the subtraction of the instrument broadening, where shape factor of “K” was used as 0.9, and the BET surface area of CeO_2_/Y_2_O_3_. No significant difference in the crystallite size of Y_2_O_3_ was observed for CeO_2_/Y_2_O_3_(CP), CeO_2_/Y_2_O_3_(HP) and CeO_2_/Y_2_O_3_(I). In accordance with this, these samples gave similar BET surface area in the range of 23–28 m^2^g^−1^. On the other hand, the BET surface area of CeO_2_/Y_2_O_3_(HT) was lower than those of other samples, although the crystallite size of Y_2_O_3_ was evaluated to be small. This conflict might be ascribed to the formation of aggregated Y_2_O_3_ crystallites with less-porous structure via hydrothermal process (Haneda et al., 2018).

**Figure 2:**
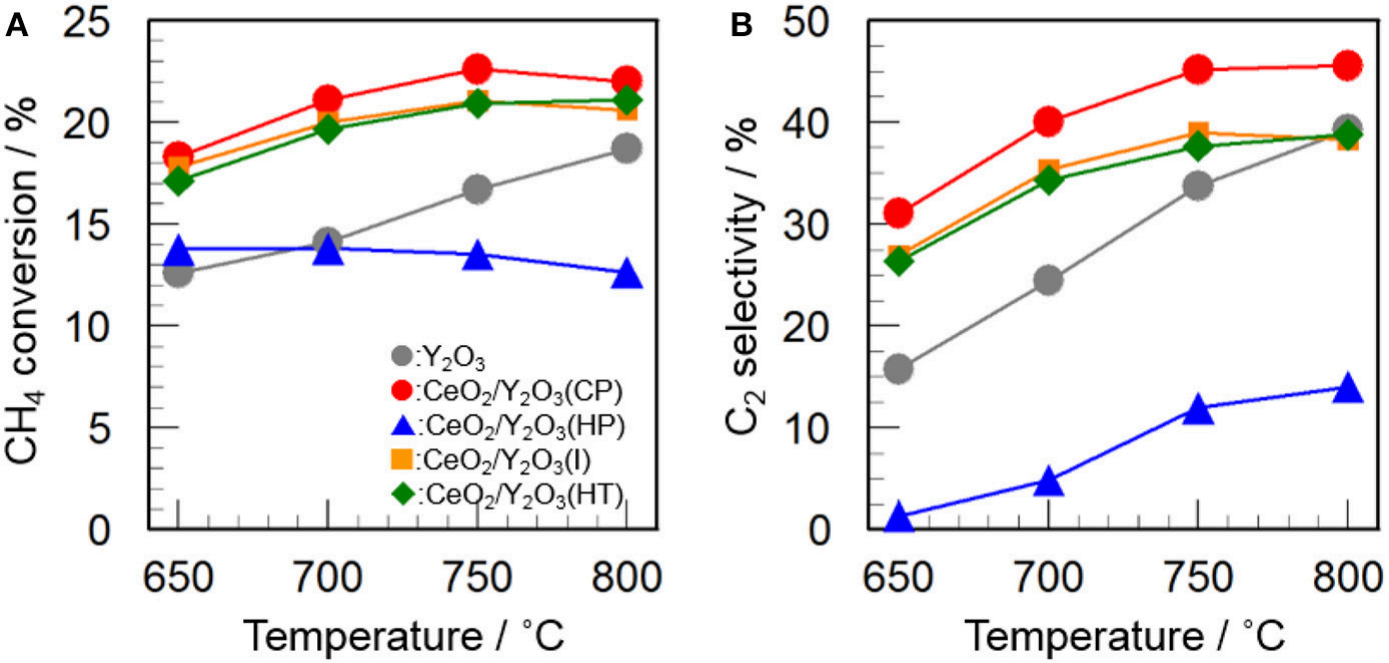
Catalytic activity of 3 mol% CeO_2_/Y_2_O_3_ for the OCM reaction. **(A)** CH_4_ conversion and **(B)** C_2_ selectivity.

Figure 2 shows the catalytic activity of 3 mol% CeO_2_/Y_2_O_3_ for the OCM reaction. It appears that the addition of CeO_2_ into Y_2_O_3_ by coprecipitation (CP), impregnation (I), and hydrothermal (HT) processes caused an increase in not only CH_4_ conversion (Figure 2A) but also C_2_ selectivity (Figure 2B) in the entire temperature range, suggesting that CeO_2_ is effective additive for the OCM reaction. Since the participation of oxygen species such as O^−^, O${}_{2}^{-}$, O^2−^, and O${}_{2}^{2-}$ formed on the catalyst surface in the activation of CH_4_ molecule for the OCM reaction has been established by many researchers (Keller and Bhasin, 1982; Borchert and Baerns, 1997; Sekine et al., 2009; Takanabe, 2012; Liang et al., 2014), active oxygen species would be efficiently supplied from the lattice of CeO_2_. On the other hand, as seen in Figure 2, CeO_2_/Y_2_O_3_(HP) showed lower CH_4_ conversion and C_2_ selectivity than Y_2_O_3_. This is probably ascribed to the formation of CeO_2_-Y_2_O_3_ solid solution revealed from XRD measurements (Figure 1Bb), resulting in ineffective supply of active oxygen species. Taking into account the fact that CeO_2_/Y_2_O_3_(HP) possesses similar physico-chemical properties, such as BET surface area and crystallite size (Table 1), with CeO_2_/Y_2_O_3_(CP) and CeO_2_/Y_2_O_3_(I), it is suspected the presence of the important factors affecting the catalytic activity of CeO_2_/Y_2_O_3_ for the OCM reaction.

### Effect of CeO_2_ loading on the activity of CeO_2_/Y_2_O_3_(CP)

#### Structural characterization of CeO_2_/Y_2_O_3_(CP)

**Figure 3:**
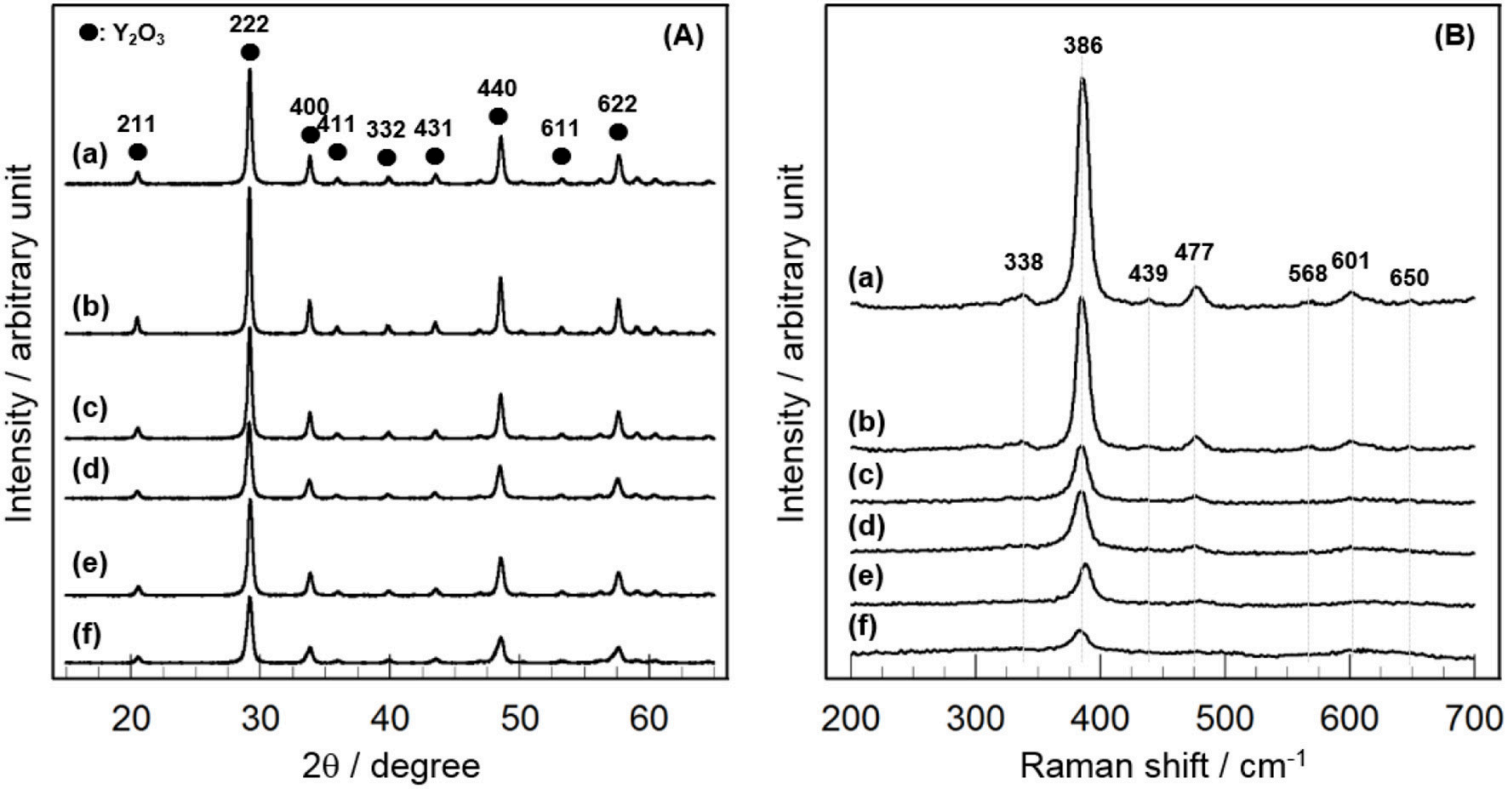
Structural characterization of CeO_2_(*x*)/Y_2_O_3_(CP) by **(A)** XRD and **(B)** Raman. (a) Y_2_O_3_, (b) CeO_2_(*1*)/Y_2_O_3_(CP), (c) CeO_2_(*3*)/Y_2_O_3_(CP), (d) CeO_2_(*4*)/Y_2_O_3_(CP), (e) CeO_2_(*5*)/Y_2_O_3_(CP), (f) CeO_2_(*10*)/Y_2_O_3_(CP).

In order to gain information on the activity controlling factor, the catalytic performance of CeO_2_(*x*)/Y_2_O_3_(CP) with different CeO_2_ loading was examined. Structural characterizations were carried out. In Table 2 are summarized the BET surface area of CeO_2_(*x*)/Y_2_O_3_(CP). It appears that no significant difference in the BET surface area was obtained in the range of 19–27 m^2^g^−1^. Figure 3A shows the XRD patterns of CeO_2_(*x*)/Y_2_O_3_(CP). No other peaks, except for those assignable to Y_2_O_3_, were detected irrespective of CeO_2_ loading. It is also noteworthy that a shift of XRD peaks by the addition of CeO_2_ was not recognized. Figure 3B shows the Raman spectra of CeO_2_(*x*)/Y_2_O_3_(CP). Raman bands ascribed to *F*_*g*_ + *A*_*g*_ mode of cubic Y_2_O_3_ with C-type structure (Repelin et al., 1995; Yashima et al., 1997; Ubaldini and Carnasciali, 2008) were observed for all the samples, and their intensities were gradually decreased with an increase in CeO_2_ loading. From XRD and Raman measurements, CeO_2_ particles were revealed to present as highly dispersion state on the surface and/or in the bulk of Y_2_O_3_.

**Table 2:** BET surface area, crystallite size of Y_2_O_3_ and the amount of H_2_ consumption in H_2_-TPR of CeO_2_(*x*)/Y_2_O_3_(CP).

	**BET surface area (m^2^g^−1^)**	**Crystallite size^a^ (nm)**	**Amount of H_2_ consumption of low-temperature peak^b^ (μmol-H_2_·g-cat^−1^)**
Y_2_O_3_	23.9	40.0	5.04
CeO_2_(*1*)/Y_2_O_3_(CP)	23.3	31.0	6.82
CeO_2_(*3*)/Y_2_O_3_(CP)	26.3	25.1	11.62
CeO_2_(*4*)/Y_2_O_3_(CP)	22.6	21.1	9.17
CeO_2_(*5*)/Y_2_O_3_(CP)	25.6	20.1	8.69
CeO_2_(*10*)/Y_2_O_3_(CP)	19.3	19.9	5.09

a *The crystallite size of Y_2_O_3_ was calculated from the XRD peak, given in Figure 1, from the (222) plane using Scherrer's equation*.

b *The amount of H_2_ consumption for the reduction of surface/subsurface oxygen species in the temperature range of 100–400°C, given in Figure 7, was estimated*.

**Figure 4:**
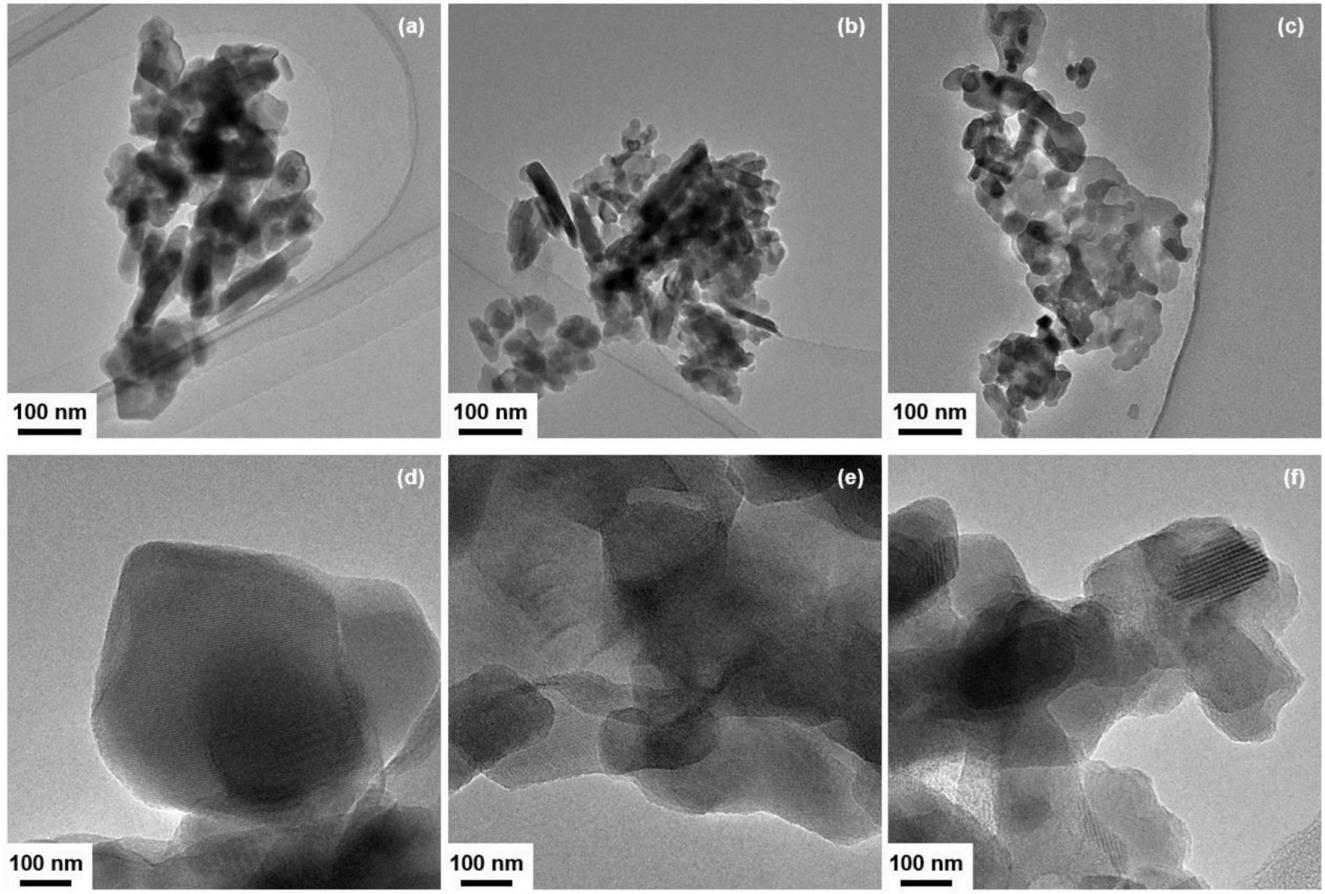
TEM images of **(a,d)** Y_2_O_3_, **(b,e)** CeO_2_(*3*)/Y_2_O_3_(CP), **(c,f)** CeO_2_(*10*)/Y_2_O_3_(CP).

In order to gain information on the dispersion state of CeO_2_ particles, the morphology of CeO_2_(*x*)/Y_2_O_3_(CP) with the CeO_2_ loadings (*x*) of 0, 3, and 10 mol% was observed by TEM analysis. As can be seen in Figures 4a–c, the addition of CeO_2_ into Y_2_O_3_ did not cause a significant change in the particle morphology, whereas the particle size was gradually decreased. This is in accordance with the crystallite size of Y_2_O_3_ (Table 2). These results suggest that the aggregated particles observed in TEM images mainly consist of Y_2_O_3_. Figures 4d–f show the TEM images of CeO_2_(*x*)/Y_2_O_3_(CP) observed at high magnification. Unfortunately, CeO_2_ and Y_2_O_3_ particles were not clearly distinguished each other because of low CeO_2_ loading (< 10 mol%) and the same crystal system (cubic). However, it appears from the comparison of Figures 4d,e that the surface morphology of Y_2_O_3_ was changed by addition of 3 mol% CeO_2_. Namely, Y_2_O_3_ consists of the particles with smooth surface (Figure 4d), whereas the surface of Y_2_O_3_ particles in CeO_2_(*3*)/Y_2_O_3_(CP) seems to be rough (Figure 4e), suggesting the surface interaction between CeO_2_ and Y_2_O_3_. In other words, CeO_2_ nanoparticles of which the size is smaller than the detection limit by TEM and XRD might be dispersed on the surface of Y_2_O_3_ particles. On the other hand, as seen in Figure 4f, CeO_2_(*10*)/Y_2_O_3_(CP) seems to be composed of a plurality of particles with different surface morphology. CeO_2_ nanoparticles might be present not only independently but also weakly interacting with Y_2_O_3_.

#### Catalytic activity of CeO_2_/Y_2_O_3_(CP) for the OCM reaction

**Figure 5:**
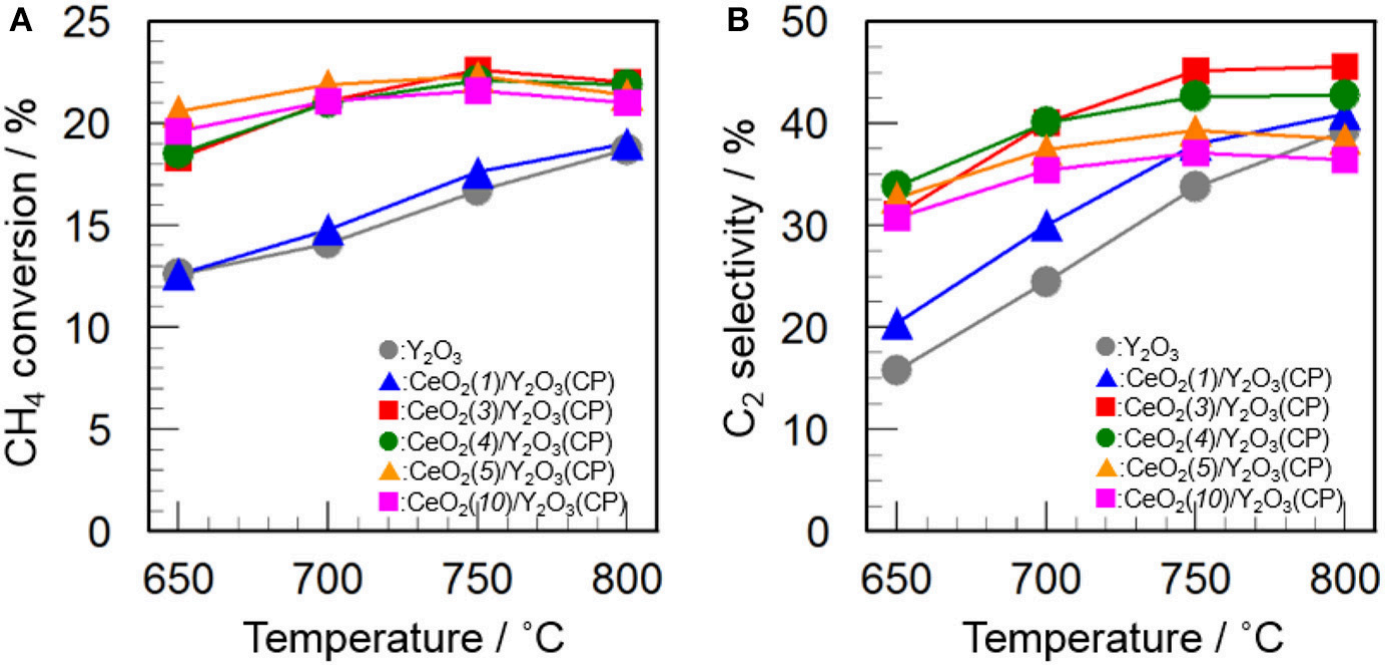
Effect of CeO_2_ loading on the activity of CeO_2_(*x*)/Y_2_O_3_(CP) for the OCM reaction. **(A)** CH_4_ conversion and **(B)** C_2_ selectivity.

Figure 5 shows the effect of CeO_2_ loading on the catalytic activity of CeO_2_(*x*)/Y_2_O_3_(CP) for the OCM reaction. As mentioned before, the addition of CeO_2_ into Y_2_O_3_ caused an increase in not only CH_4_ conversion but also C_2_ selectivity in the entire temperature range. As can be seen in Figure 5A, CH_4_ conversion was drastically increased by addition of 3 mol% CeO_2_. Further increase in CeO_2_ loading up to 10 mol% did not cause any change in the CH_4_ conversion. In accordance with previous reports (Primet and Garbowski, 2002), CeO_2_ seems to be good additive for the activation of CH_4_ molecules. On the other hand, as seen in Figure 5B, the effect of CeO_2_ loading on the C_2_ selectivity was slightly different from that on the CH_4_ conversion. The C_2_ selectivity was gradually increased with an increase in the CeO_2_ loading up. The maximum C_2_ selectivity was achieved at 3 mol% CeO_2_ loading. However, further increase in CeO_2_ loading caused a gradual decrease in the C_2_ selectivity. The optimum CeO_2_ loading for the formation of C_2_H_6_/C_2_H_4_ as a product was found to be 3 mol%. As seen in Table 2, the highest BET surface area was obtained for CeO_2_(*3*)/Y_2_O_3_(CP). This is in agreement with the catalytic activity. However, although CeO_2_(*5*)/Y_2_O_3_(CP) was found to possess similar BET surface area with CeO_2_(*3*)/Y_2_O_3_(CP), the former catalytic activity was clearly lower than that of the latter catalyst. This suggests that the BET surface area is not only the important factor to determine the catalytic activity.

**Figure 6:**
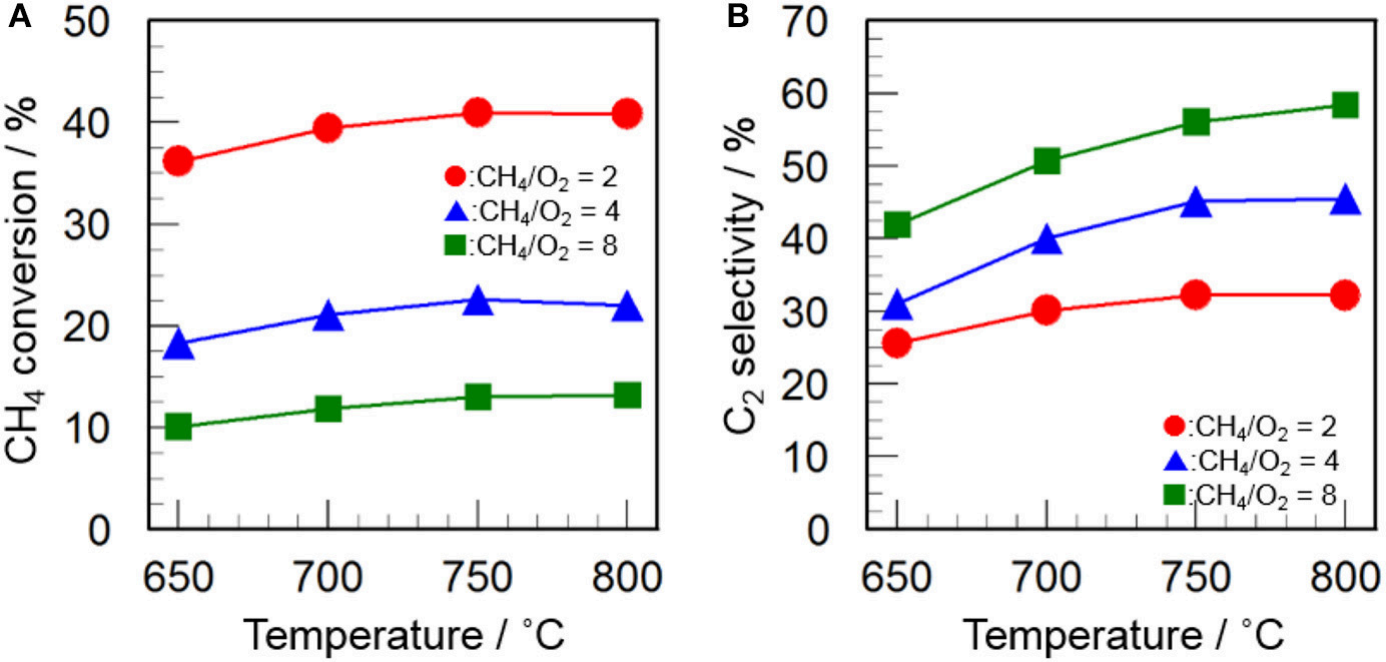
Effect of CH_4_/O_2_ ratio on **(A)** CH_4_ conversion and **(B)** C_2_ selectivity in the OCM reaction over CeO_2_(*3*)/Y_2_O_3_(CP). (Red circles) CH_4_/O_2_ = 2, (blue triangles) CH_4_/O_2_ = 4, (green squares) CH_4_/O_2_ = 8.

Since the CH_4_/O_2_ ratio is an important parameter to optimize the reaction conditions, the effect of CH_4_/O_2_ ratio on the catalytic activity of CeO_2_(*3*)/Y_2_O_3_(CP) was examined. As can be seen in Figure 6, an increase in the CH_4_/O_2_ ratio caused a decrease in the CH_4_ conversion as well as an increase in the C_2_ selectivity. This indicates that the reaction gas containing higher concentration of O_2_ is favored for the activation of CH_4_, resulting in high CH_4_ conversion, while is also favored for the formation of undesirable CO_2_, leading to low C_2_ selectivity. The trade-off between CH_4_ conversion and C_2_ selectivity is well-known phenomena in the OCM reaction (Ghose et al., 2014; Godini et al., 2014; Ivanov et al., 2014). From the kinetic point of view, the activated CH_4_ molecule (CH_3_· radical) may preferentially react with O_2_ species in the presence of excess O_2_. It would be important to explore the optimum reaction conditions to achieve high C_2_ productivity.

#### Active sites for the OCM reaction over CeO_2_/Y_2_O_3_(CP)

**Figure 7:**
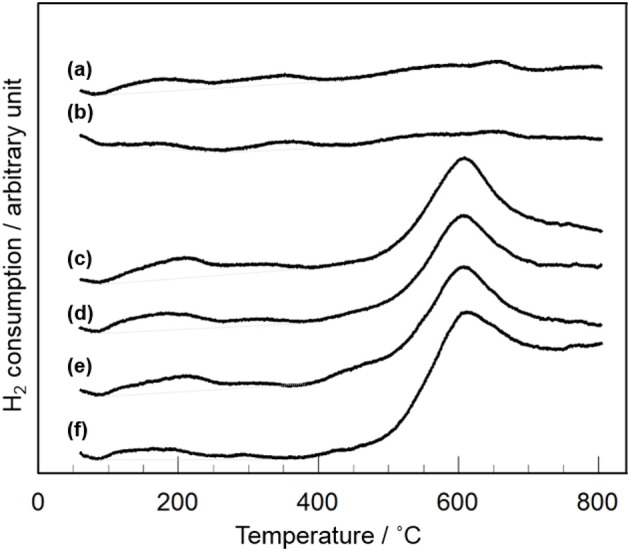
H_2_-TPR profiles of CeO_2_(*x*)/Y_2_O_3_(CP) with different CeO_2_ loading. (a) Y_2_O_3_, (b) CeO_2_(*1*)/Y_2_O_3_(CP), (c) CeO_2_(*3*)/Y_2_O_3_(CP), (d) CeO_2_(*4*)/Y_2_O_3_(CP), (e) CeO_2_(*5*)/Y_2_O_3_(CP), (f) CeO_2_(*10*)/Y_2_O_3_(CP).

XRD and Raman measurements revealed that CeO_2_ particles are present as highly dispersion state on the surface and/or in the bulk of Y_2_O_3_ (Figure 3). In addition, TEM observation suggested that the dispersion state of CeO_2_ particles is different depending on CeO_2_ loading (Figure 4). In case of CeO_2_(*3*)/Y_2_O_3_(CP), CeO_2_ nanoparticles might be dispersed on the surface of Y_2_O_3_ particles, while CeO_2_ nanoparticles might be present not only independently but also weakly interacting with Y_2_O_3_ in CeO_2_(*10*)/Y_2_O_3_(CP). Therefore, the dispersion state of CeO_2_ would be related to the catalytic activity. It is well known that the reduction of CeO_2_ particles gradually proceeds from surface to bulk in the wide range from low to high temperature region, respectively (Yao and Yao, 1984; Trovarelli, 1996; Imagawa et al., 2011). In order to obtain an information on the dispersion state of CeO_2_, H_2_-TPR measurements were carried out. As given in Figure 7a, very broad H_2_ consumption peaks in the temperature range of 100–700°C were observed in the H_2_-TPR profile of Y_2_O_3_. This is probably due to the reduction of oxygen species interacting with the defects sites in the lattice of Y_2_O_3_ with C-type structure. No significant change in the H_2_-TPR profile was observed for CeO_2_(*1*)/Y_2_O_3_(CP) (Figure 7b). This is in agreement with the tendency of the catalytic activity (Figure 5). It is noteworthy that the addition of 3 mol% CeO_2_ caused an appearance of distinct H_2_ consumption peaks at around 200 and 600°C (Figure 7c). The area of H_2_ consumption peak at around 600°C, which can be ascribed to the reduction of oxygen species in the bulk of CeO_2_ (Yao and Yao, 1984; Trovarelli, 1996; Imagawa et al., 2011), was gradually increased with an increase in CeO_2_ loading up to 10 mol%. In contrast, the low-temperature peak at around 200 °C due to the reduction of surface/subsurface oxygen species on CeO_2_ particles (Yao and Yao, 1984; Trovarelli, 1996; Imagawa et al., 2011) was clearly decreased for CeO_2_(*10*)/Y_2_O_3_(CP). These results suggest that the size of CeO_2_ particles dispersed on the surface and/or in the bulk of Y_2_O_3_ was significantly increased with an increase in CeO_2_ loading.

**Figure 8:**
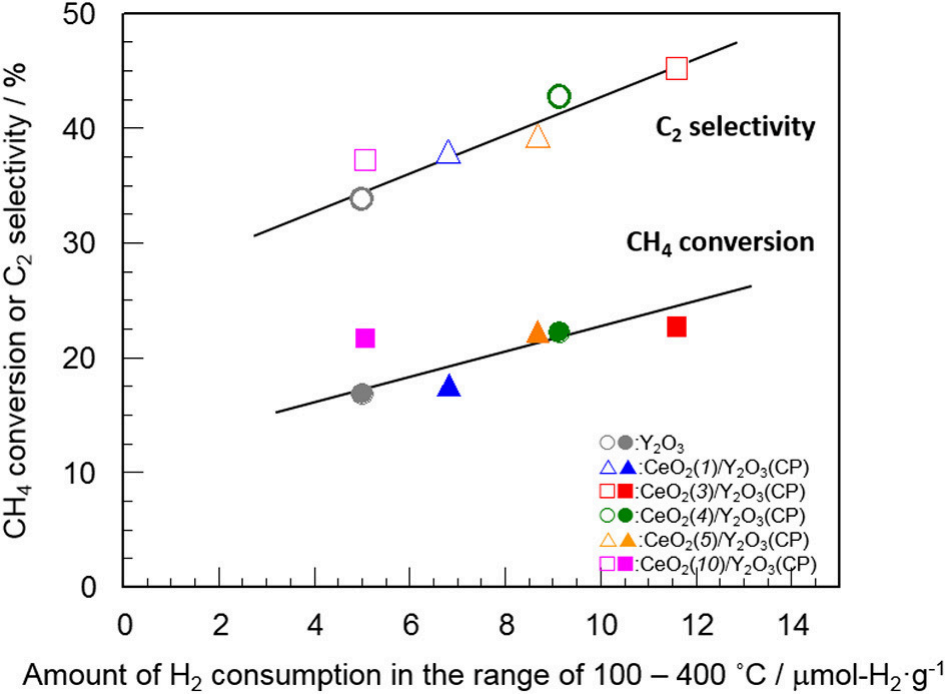
Relationship between the amount of H_2_ consumption for the reduction of surface/subsurface oxygen species and the catalytic activity for the OCM reaction at 750°C.

Table 2 summarizes the amount of H_2_ consumption for the reduction of surface/subsurface oxygen species in the temperature range of 100–400°C. It is of interest that the maximum amount of H_2_ consumption was obtained for CeO_2_(*3*)/Y_2_O_3_(CP), and then decreased with CeO_2_ loading. Figure 8 shows the relationship between the amount of H_2_ consumption for the reduction of surface/subsurface oxygen species and the catalytic activity for the OCM reaction at 750 °C. It appears that the CH_4_ conversion and C_2_ selectivity were linearly increased with an increase in the amount of H_2_ consumption. This clearly indicates that oxygen species on the surface/subsurface of highly dispersed CeO_2_ particles can effectively activate CH_4_ molecule, resulting in the selective formation of C_2_H_6_/C_2_H_4_. On the basis of XRD, Raman, TEM, and H_2_-TPR measurements, we can conclude that the creation of highly dispersed CeO_2_ particles is one of the strategies for the catalyst design leading to the development of highly active OCM catalysts.

### Catalytic role of CeO_2_ in the OCM reaction

Since CeO_2_ is well-known material showing reversible oxygen release/absorb properties (Trovarelli, 1996), the ^16^O/^18^O isotopic exchange reaction was carried out to clarify the possibility of O_2_ activation as catalytically role of CeO_2_ in the OCM reaction. Martin and Duprez (1996) and Duprez (2006) investigated the oxygen mobility of various kinds of oxide by using the ^16^O/^18^O isotopic exchange reaction, and proposed two mechanisms depending on the type of oxide. One group is non-reducible oxide such as Al_2_O_3_, SiO_2_, ZrO_2_ and MgO, and the other is reducible oxide such as CeO_2_ and CeO_2_-ZrO_2_. In the former case, the simple hetero-exchange that occurs with the participation of only one oxygen of the oxide at each step of the following equations (1) and (2):

(1) $${}^{18}{\text{O}}_{2}\left(\text{g}\right){+}^{16}\text{O}\left(\text{s}\right)\;\to^{18}{\text{O}}^{16}\text{O}\left(\text{g}\right){+}^{18}\text{O}\left(\text{s}\right)$$

(2) $${}^{18}{\text{O}}^{16}\text{O}\left(\text{g}\right){+}^{16}\text{O}\left(\text{s}\right)\;\to^{16}{\text{O}}_{2}\left(\text{g}\right){+}^{18}\text{O}\left(\text{s}\right)$$

Here, the consecutive evolution of ^18^O^16^O and ^16^O_2_ can be observed. On the other hand, the isotopic exchange reaction over reducible oxides takes place *via* the multiple-hetero exchange mechanism, where the reaction between a molecule dioxygen (^18^O_2_) in gas phase and two atomic oxygens (^16^O) of the solid occurs the following equation (3) or (4):

(3) $${}^{18}{\text{O}}^{16}\text{O}\left(\text{g}\right){+}^{16}\text{O}\left(\text{s}\right)\;\to^{16}{\text{O}}_{2}\left(\text{g}\right){+}^{18}\text{O}\left(\text{s}\right)$$

(4) $${}^{18}{\text{O}}_{2}(\text{g})+2^{16}\text{O}(\text{s})\;\to^{16}{\text{O}}_{2}(\text{g})+2^{18}\text{O}(\text{s})$$

In this case, ^16^O_2_ and ^16^O^18^O seem to be simultaneously evolved.

**Figure 9:**
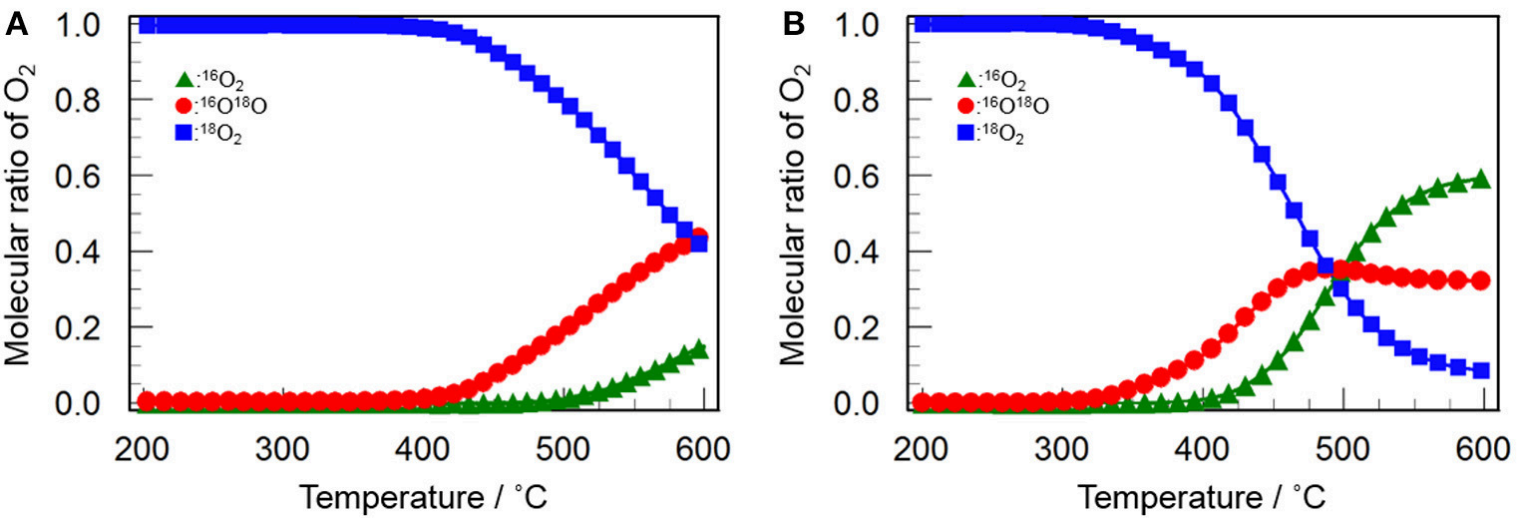
Change in the molecular ratio of ^16^O_2_ (green triangles), ^16^O^18^O (red circles) and ^18^O_2_ (blue squares) during the isotopic exchange reaction of O_2_ over **(A)** Y_2_O_3_ and **(B)** CeO_2_(*3*)/Y_2_O_3_(CP) as a function of temperature.

Figure 9 shows the profiles of ^16^O_2_ and ^16^O^18^O evolution and ^18^O_2_ consumption in the ^16^O/^18^O isotopic exchange reaction over Y_2_O_3_ and CeO_2_(*3*)/Y_2_O_3_(CP). It appears that both catalysts gave similar profiles for each O_2_ species. The evolution of ^16^O^18^O and the consumption of ^18^O_2_ were simultaneously observed, followed by the ^16^O_2_ evolution at higher temperature. This suggests that the simple hetero-exchange mechanism is favored on Y_2_O_3_ and CeO_2_/Y_2_O_3_ with lower CeO_2_ loading, although the O_2_ activation over CeO_2_-containing sample was suspected to be governed by the multiple-hetero exchange mechanism. This is probably due to the high dispersion state of CeO_2_ nanoparticles on the surface of Y_2_O_3_, as revealed by H_2_-TPR measurement (Figure 7).

Dong et al. (2004a,b) investigated the oxygen mobility of CeO_2_-ZrO_2_ based catalyst with different structural homogeneity, and reported that oxygen species activated at lower temperature *via* the multiple-hetero exchange mechanism is highly active for the complete oxidation reaction. It can be expected that oxygen species with moderate activity, which was evolved *via* the simple hetero-exchange mechanism, plays a beneficial role in CH_4_ activation (Kumar et al., 2016), leading to the selective oxidation to C_2_H_6_/C_2_H_4_. Taking into account the fact that the temperature for the evolution of ^16^O^18^O and ^16^O_2_ was significantly lowered by addition of CeO_2_ into Y_2_O_3_ (Figure 9), we can conclude that the catalytically role of CeO_2_ in the OCM reaction is to promote the formation of active oxygen species *via* the simple hetero-exchange mechanism.

## Conclusion

The catalytic activity of Y_2_O_3_ for the OCM reaction was effectively improved by addition of CeO_2_. Among the catalysts tested here, CeO_2_/Y_2_O_3_ prepared by coprecipitation method showed the highest activity. The optimum CeO_2_ loading was 3 mol%. From the structural characterizations by XRD, Raman and TEM, CeO_2_ particles were found to be dispersed on the surface of Y_2_O_3_ without the formation of CeO_2_-Y_2_O_3_ solid solution. H_2_-TPR measurements revealed that the amount of H_2_ consumption for the reduction of CeO_2_ surface was increased with an increase in CeO_2_ loading and then reached the maximum at 3 mol%. Highly dispersed CeO_2_ particles was suspected to act as catalytically active sites in the OCM reaction. From the ^16^O/^18^O isotopic exchange reaction, the beneficial role of CeO_2_ in the OCM reaction was concluded to promote the formation of active oxygen species *via* the simple hetero-exchange mechanism.

## Author contributions

MH: worked on the experimental setup; YK and YN: conducted the experiments; AT: carried out the TEM analysis.

### Conflict of interest statement

The authors declare that the research was conducted in the absence of any commercial or financial relationships that could be construed as a potential conflict of interest.
